# Early telemedicine training and counselling after hospitalization in patients with severe chronic obstructive pulmonary disease: a feasibility study

**DOI:** 10.1186/s12911-014-0124-4

**Published:** 2015-02-07

**Authors:** Lisbeth Rosenbek Minet, Line Willads Hansen, Claus Duedal Pedersen, Ingrid Louise Titlestad, Jette Krøjgaard Christensen, Kristian Kidholm, Kathrine Rayce, Alison Bowes, Lilian Møllegård

**Affiliations:** Institute of Clinical Research, Faculty of Health Sciences, University of Southern Denmark, JB Winsløws Vej 19, 5000 Odense C, Denmark; Department of Rehabilitation, Odense University Hospital, Sdr. Boulevard 29, 5000 Odense C, Denmark; Department of Clinical Innovation, Odense University Hospital, Sdr. Boulevard 29, 5000 Odense C, Denmark; Department of Respiratory Medicine, Odense University Hospital, Sdr. Boulevard 29, 5000 Odense C, Denmark; Department of Medicine, Odense University Hospital, Svendborg Hospital, Valdemarsgade 53, 5700 Svendborg, Denmark; Department of Research and Health Technology Assessment, Odense University Hospital, Sdr. Boulevard 29, 5000 Odense C, Denmark; School of Applied Science, University of Stirling, FK9 4LA Stirling, Scotland UK

**Keywords:** Pulmonary rehabilitation, Training, Counselling, Telemedicine, Videoconference, COPD, Chronic disease, Internet

## Abstract

**Background:**

An essential element in the treatment of patients with chronic obstructive pulmonary disease (COPD) is rehabilitation, of which supervised training is an important part. However, not all individuals with severe COPD can participate in the rehabilitation provided by hospitals and municipal training centres due to distance to the training venues and transportation difficulties. The aim of the study was to assess the feasibility of an individualized home-based training and counselling programme via video conference to patients with severe COPD after hospitalization including assessment of safety, clinical outcomes, patients’ perceptions, organisational aspects and economic aspects.

**Methods:**

The design was a pre- and post-test intervention study. Fifty patients with severe COPD were included. The telemedicine training and counselling included three weekly supervised exercise sessions by a physiotherapist and up to two supervised counselling and training sessions in energy conservation techniques by an occupational therapist. The telemedicine videoconferencing equipment was a computer containing a screen, a microphone, an on/off switch and a volume control.

**Results:**

Thirty seven (74%) participants completed the programme, with improvements in health status assessed by the Clinical COPD Questionnaire and physical performance assessed by a sit-to-stand test and a timed-up-and-go test. There were no cases of patient fall or emergency contact with a general practitioner during the telemedicine training sessions. The study participants believed the telemedicine training and counselling was essential for getting started with being physically active in a secure manner. The business case showed that under the current financing system, the reimbursement to the hospital was slightly higher than the hospital expenditures. Thus, the business case for the hospital was positive. The organizational analysis indicated that the perceptions of the staff were that the telemedicine service had improved the continuity of the rehabilitation programme for the patients and enabled the patients’ everyday lives to be included in the treatment.

**Conclusions:**

This study showed that home-based supervised training and counselling via video conference is safe and feasible and that telemedicine can help to ensure more equitable access to supervised training in patients with severe COPD.

**Trial registration:**

Clinical Trials NCT02085187 (Date of registration 10.03.2014).

## Background

Chronic obstructive pulmonary disease (COPD) is a common chronic disease in Denmark and worldwide. It is estimated that approximately 3 million people die of COPD every year, and that about 400,000 people in Denmark have COPD [1]. COPD is a progressive disease, with symptoms including shortness of breath, coughing and expectoration. Some patients experience exacerbations which are often due to infections of the respiratory tract, requiring change in medication and, in severe cases, hospitalization. Many people with COPD experience difficulties that affect their everyday lives. An essential non-pharmacological element in the treatment of COPD is rehabilitation, of which therapeutically supervised training is an important part. There is evidence that rehabilitation of patients with COPD in a stable phase, relieves dyspnoea and fatigue, improves emotional function and enhances the patients’ sense of control over their condition [2]. Rehabilitation, including physiotherapy mobilization and training, is recommended for patients with COPD in an acute phase who are hospitalized with exacerbation of COPD, and should continue after discharge, as it has been shown to reduce readmission, mortality and increase health related quality of life [3]. It is recommended that patients with a Medical Research Council (MRC) Dyspnoea score of 3–5 who are functionally limited by breathlessness are offered pulmonary rehabilitation [4] and that pulmonary rehabilitation is commenced within 4 weeks after exacerbation of COPD [5].

Despite evidence of the importance of rehabilitation in disease process and management, there are still barriers associated with participation in established rehabilitation programmes provided by hospitals and municipal training centres [6]. The main reasons for non-participation in rehabilitation programmes are disruption of daily routines, distance to the training venues and transportation difficulties [6]. Thus, it is important to develop pulmonary rehabilitation that involves physical training, to overcome these barriers without serious impact on the patient’s resources and income. Telemedicine, which makes long distance communication with patients possible, could ideally be used to offer supervised training in their own home.

Previous research has demonstrated that various forms of telemedicine are feasible, but there is a lack of information about what effects they have on health outcomes and costs [7], and the knowledge about the use of telemedicine strategies in pulmonary rehabilitation is limited [8]. Nevertheless there is a growing interest in using health technologies to provide safe and effective telemedicine solutions involving supervised training for people with COPD. A center-based telemedicine rehabilitation programme involving supervised training was found to be an effective tool for increasing COPD pulmonary rehabilitation [9]. However this telemedicine strategy did not overcome transportation difficulties. Another study examining the feasibility of a long-term telerehabilitation service involving training at home for COPD patients suggests that it may reduce healthcare utilization [10]. A recently study found that home-based supervised training using existing widely available technology is safe and feasible for people with COPD [11]. This study found that effective delivery of telerehabilitation requires an adequate data network. Further research is needed to show whether supervised training via telemedicine is feasible and effective in patients with severe COPD after hospitalization with exacerbation.

At present, patients with severe COPD hospitalized at Odense University Hospital with exacerbation of COPD are offered rehabilitation including training in the hospital after being discharged. Some of these patients decline participation in the COPD rehabilitation due to transportation difficulties. To make sure that all patients hospitalized with exacerbation of COPD have access to supervised training after discharge, the aim of the present study was to evaluate the feasibility of an individualized home based training and counselling programme delivered via video conference to patients with severe COPD. It examined safety, clinical outcomes, patients’ perceptions, economic and organisational aspects. Thus, this feasibility study includes most domains in the multidisciplinary model for assessment of telemedicine MAST [12]. MAST is a model or framework for assessment of telemedicine applications that is based on a service provider’s need for information for decision making on whether to implement telemedicine. MAST includes 7 assessment domains (health problem and characteristics of the application, safety, clinical effectiveness, patient perspectives, economic aspects, organisational aspects, and socio-cultural, ethical and legal aspects).

## Methods

### Design

Patients were enrolled consecutively from 1 January 2012 to 30 December 2012. Patients who agreed to participate in the project were contacted via video conference by a physiotherapist one week after discharge and immediately after the first week’s counselling with the nurse had been completed. The intervention lasted for 3 weeks. All the patients included were studied with regard to health-related outcomes, pre-intervention (at the beginning of the intervention programme) and post intervention (at the end of the programme). A survey of the patients’ subjective perceptions of the programme was carried out post intervention.

### Settings

This study was conducted at the Department of Respiratory Medicine at Odense University Hospital in Denmark. The hospital has an intake of patients from both urban and rural areas of the region. All patients with exacerbation of COPD requiring hospitalization are admitted the Emergency Department and transferred to the Department of Respiratory Medicine. Usual care for patients with severe COPD is twice weekly participation for 8 weeks in an out-patient hospital based rehabilitation programmes that include supervised exercise training and patient education. For patients hospitalized with acute exacerbation of COPD the usual care approach also includes telemedicine education sessions with a nurse daily in the first week after discharge focusing on lung health, drugs, nutrition, coping and stress management [13].

### Participants

Inclusion criteria for patients were: severe and very severe COPD, i.e. with an FEV_1_ value under 50% of the predicted value [14], an FEV_1_/FVC ratio < 70% [14], MRC (Medical Research Council Dyspnoea Scale) grade 3–5 [15], age ≥40 years, hospitalization with exacerbation of COPD, declined participation in the hospital-based rehabilitation and participation in videoconference sessions with a nurse for one week immediately after discharge. Patients were included in the study straight after the videoconference with the nurse during the first week after discharge from hospital.

The exclusion criteria were: inability to communicate via telephone and computer; systolic BP <100 mm Hg; X-rays of the thorax showing abnormalities suspicious of thoracic malignancy or lobar pneumonia; a diagnosis of cancer or recurrence of cancer within the last 5 years; hospitalization with septic shock, acute myocardial infarction (AMI) or other serious medical conditions (e.g. kidney disorder); heart failure (EF < 30%), or if the patient did not wish to participate.

### The equipment

**Figure 1:**
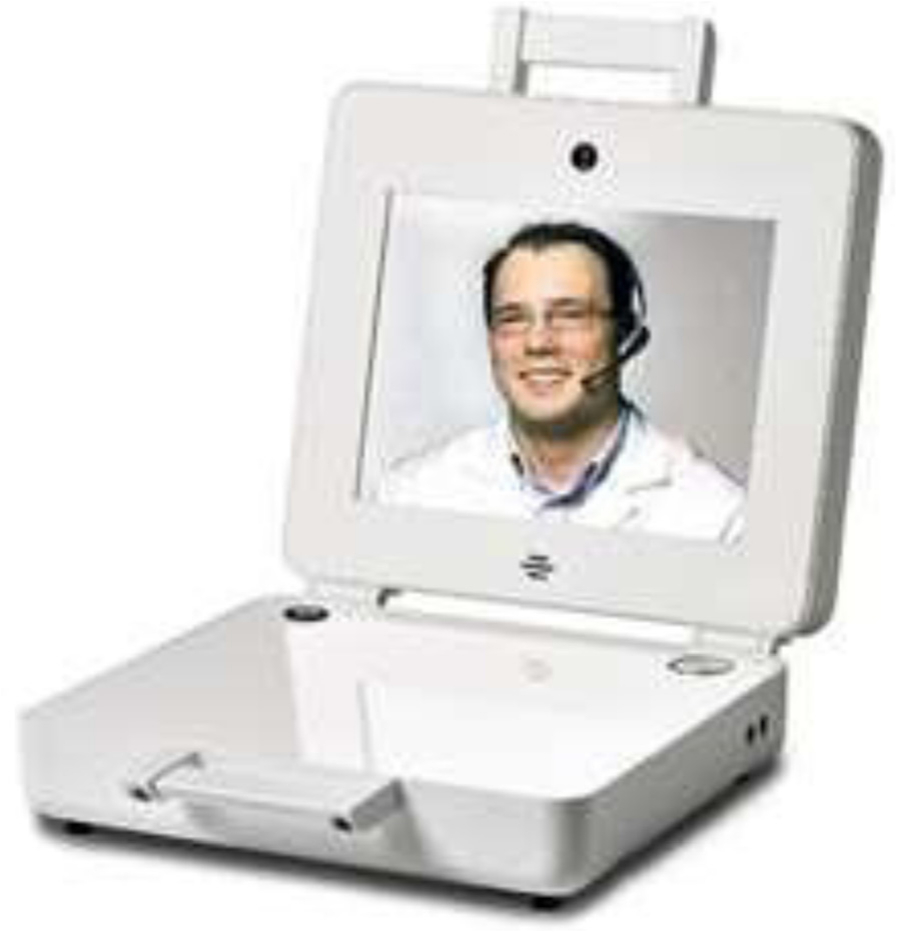
**The telemedicine equipment “Patient Briefcase” placed in the patient’s own home.**

**Figure 2:**
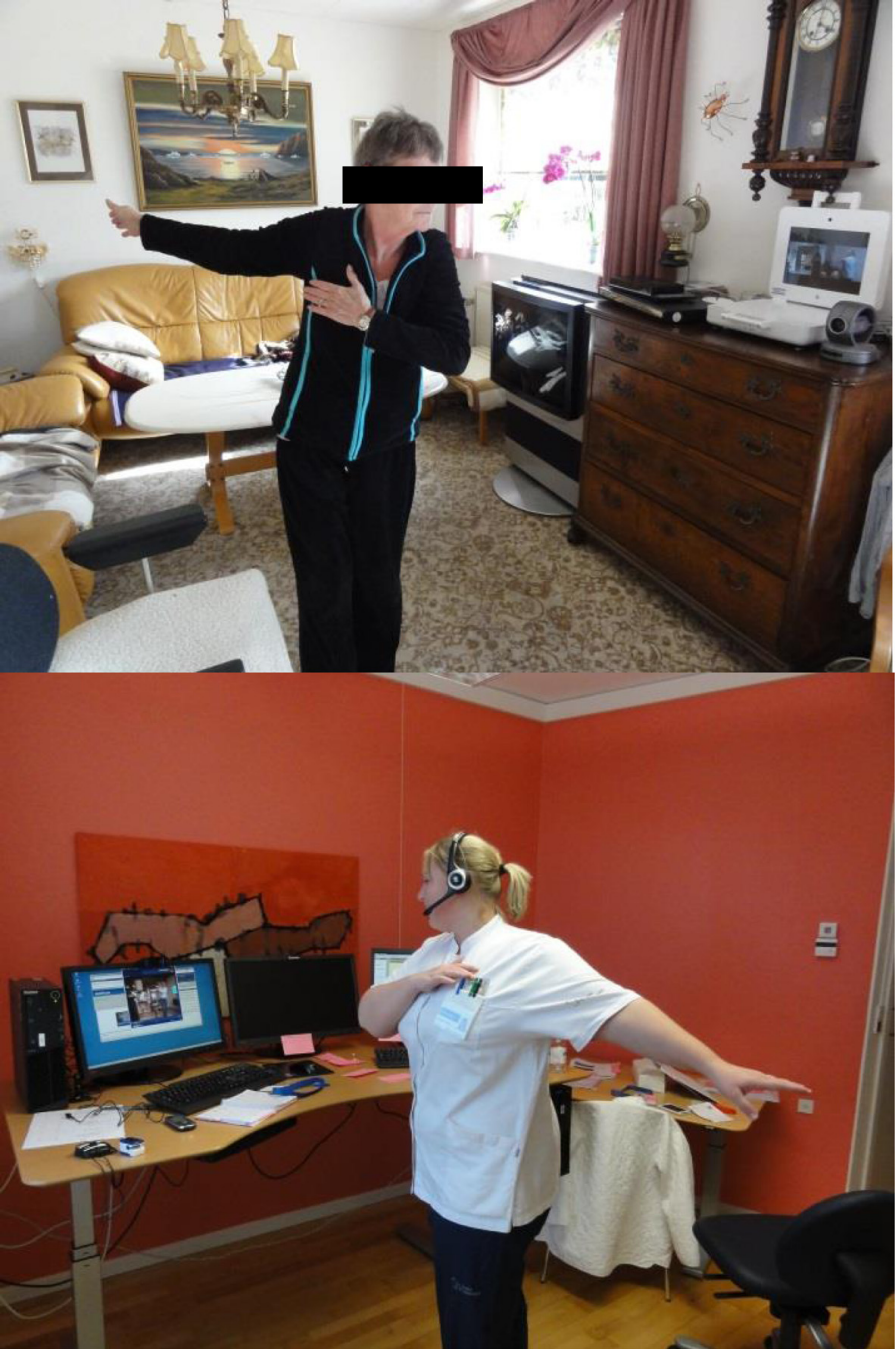
**Training via video conference.** The patients are trained in their own home supervised by a physiotherapist located at the hospital.

The telemedicine videoconferencing equipment was designed to look like a briefcase and was known as the “Patient Briefcase”. The briefcase contained a screen, microphone, an on/off switch and a volume control (Figure 1). A camera was installed in the patient’s home together with the briefcase. The camera made it possible to follow the patients’ movements around the room during the training session. A pulse oximeter was used to monitor saturation and heart rate. The therapist’s equipment consisted of a computer with an in-built webcam and microphone, a second display for reading patient measurements and a computer for the electronic patient record. Communication took place via the internet (ADSL), wireless or satellite communication, and measurements were transferred from the patient’s home to the hospital in a closed secure system. Thus only invited users could participate in, view or hear the videoconferences. The patient’s equipment was installed by a technician who also provided instruction in how to switch the system on and off and how to position the fingerclip pulse oximeter. Figure 2 shows the supervised training via the Patient Briefcase.

### The intervention

The intervention consisted of training and counselling sessions with respectively a physiotherapist and an occupational therapist.

Training and counselling by the physiotherapist consisted of 3 weekly sessions, lasting 30–45 minutes, over a 3 week period, i.e. a total of 9 supervised sessions. The telemedicine training was individualized and based on participant’s needs and goals. Heart rate and oxygen saturation were monitored during the exercise training for safety. The training was based on recommendations from Zainuldin [16] and Holland et al. [17] and included thoracic mobilization exercises, cardio training with an intended intensity of 60%-90% of max capacity, strength training with an intended intensity of 60% of 1 Repetitions Maximum (RM) and breathing exercises. The training involved: 5–10 minutes warming up, consisting of thoracic mobilization exercises, exercises for upper extremities, lower extremities and neck/shoulder; 10–15 minutes cardio training, consisting of swing exercises, walking on the spot, seated exercises and, if possible, a stair workout; 10–15 minutes strength training, consisting of elastic exercises for upper and lower extremities, standing squats and stand and sit chair exercises. Breathing exercises such as pursed lip breathing and diaphragmatic breathing were used between the exercises to alleviate intercostal breathing. The training intensity was continuously progressed. In addition, patients were asked to train on their own on days when there was no telemedicine session with a physiotherapist.

There were 1–2 sessions with the occupational therapist, which consisted of training and counselling on energy conservation techniques. The first session was 60 minutes long and included assessment, counselling and training. This session was delivered in the second week of the intervention. If required, a second session of 30 minutes was given in the third week. The counselling and training in energy conservative techniques [18] was based on assessing the patient’s problem areas of occupational performance and patient prioritization of their daily activities using The Canadian Occupational Performance Measure (COPM) [18].

### Measurements

All descriptive characteristics and medical information were extracted from the medical records.

Safety was assessed by registration of patients’ falls during the patient training and need for acute contact to the general practitioner. Information about safety was assessed from the patients’ medical records.

Clinical outcomes were estimated as changes in health status and physical performance. Health status was assessed by the Clinical COPD Questionnaire (CCQ), a self-administered validated questionnaire measuring the health status of patients with COPD [19]. Studies [20] have shown that a mean change in CCQ of 0.4 in the total score constitutes a minimum relevant clinical change. Physical performance was estimated as the base mobility and strength in the lower limb using two validated functional tests. The outcome measures of mobility were firstly Timed Up and Go test (TUG), measuring the time in seconds (with 2 places), it takes a person to rise from an ordinary chair (with back and armrest), walk 3 meters, turn around and walk back to the chair and sit down again [21]. Secondly, the five times sit-to-stand test (FTSST) was used to measure functional lower limb muscle strength, where the time in seconds (2 places) used to rise 5 times from a chair is measured [22]. The functional tests were carried out by specially trained therapists and were performed in the patient’s home just before and just after the telemedicine training and counselling. At the time of the assessment the patients were in the sub-acute phase.

Patients’ subjective perceptions were assessed by asking them to submit a postcard with information about their experience. The postcard was an open ended question and was printed with the text “Dear Department of Rehabilitation. My experience of training and counselling using the Patient briefcase was … ”.

The assessment of the economic aspects was done in a business case including expenditures per patient participating in the telemedicine rehabilitation programme and reimbursement to the hospital. Thus, the business case included only on economic impact and financing for the hospital and a societal cost-effectiveness (SCE) analysis was not conducted. The need for SCE information in successful implementation has been stressed [23]. Since the patients involved had declined to participate in supervised training at the hospital, there were no expenditures of ‘usual care’ with regard to rehabilitation. Expenditures included renting of the “patient briefcase”, establishment of a safe line for data transmission and use of hospital staff. Reimbursement was estimated as the DRG (Diagnosis Related Groups) value of the rehabilitation activity. This is similar to the actual payment for the activity from the regional health system (The Region of Southern Denmark) to the hospital in accordance with the health care financing system. The use of staff was assessed during an interview with the project manager, who carried out the telemedicine project. Information on reimbursement was collected by recording of the length of use of the “patient briefcase” from the Department of Planning and Data at the hospital. Prices for renting of the “patient briefcase” etc. are described in a contract between the producer (Medisat) and the hospital and the content is confidential. Therefore prices cannot be presented here. Instead the total expenditures and the quantities of resource use are described.

Hospital staff perceptions of the telemedicine intervention programme were assessed by a focus group interview with occupational - and physiotherapists who carried out the telemedicine rehabilitation training and thus were familiar with the programme. The interview was conducted by a researcher not involved in the programme delivery to facilitate unbiased data collection. During the focus group interview, participants were asked for their views on five topics that were considered important based on a previous study of the technology [24]: the technical reliability of the service; patient safety; organization of the telemedicine service at the hospital; collaboration with primary care; and the contact to the patients by use of the service. Similar methods haves been used in other studies of organizational aspects of telemedicine, e.g. see Taylor et al. [25].

### Data analysis

Data on health status and physical performance were not normally distributed and a non-parametric test (related-samples Wilcoxon Signed Ranks Test) was used to compare changes in CCQ, TUG and FTSST pre-intervention and post intervention. The results are presented as medians (25th percentile;75th percentile). A p-value of < 0.05 was regarded as statistically significant. For data analyses, SPSS (version 20, Chicago, IL, USA) for Windows was used. A per-protocol analysis was performed and restricted to the participants who fulfil the protocol in the terms of the eligibility, interventions, and outcome assessments.

The data obtained on patient perceptions was analysed using thematic content analysis. The purpose was to identify the main themes in the participants’ accounts of their experiences with the intervention. This is a comparative process [26], by which the various accounts gathered from the postcard were compared with each other to classify themes that were common or recurrent in the data set. A word processor Microsoft Word version 2010 was used to produce a table in which the data set was cut up and rearranged under thematic headings. The first author carried out the thematic analysis. The focus groups were audio taped and transcribed. Themes were identified using codes and creating categories. To validate the results, the interpretation was shared with the respondents afterwards for approval.

### Research ethics

The protocol for this study was approved by the Regional Scientific Ethics Committee of Southern Denmark (project-ID: S-20110036) as a development project and the study was reported to the Danish Data Protection Agency. The study was conducted in compliance with all national regulations governing the protection and privacy of human subjects and the Helsinki Declaration. Interested participants who met the inclusion criteria were requested informed consent. Once written informed consent was obtained from the participants they were included in the study. Written informed consent was obtained from the participants to publish their image.

## Results

Fifty people were included in the study during the period 1 January to 1 December 2012. Of these 37 (74%) patients completed a full intervention programme and 13 patients withdrew during the course of the programme. Reasons for dropouts were as follows: seven were hospitalised, four had worsening COPD or other diseases and two declined to participate. Patients who carried out a full programme had on an average 7.5 video links with physiotherapist, with average time per session of 36 minutes. All patients had at least one telemedicine session with the occupational therapist (average duration 50 minutes), 10 patients (=20%) needed the additional second session with the occupation therapist. Baseline data for the 37 patients completing the programme are presented in Table 1.

**Table 1:** **Baseline demographic characteristics of patients completing the intervention**

**Patients n = 37**	**Mean/number**	**Standard deviation/per cent**
Age (year)	69.2	8.8
Gender (Men)	5	14%
Living alone	20	54%
Current prednisolone treatment	14	38%
FEV1 (%)	27.1	12.5
MRC	4.5	0.7
BMI (kg/m2)	23.0	5.0
Borg, in rest	2.6	1.4
Long term oxygen therapy (n)	17	46%
Saturation, in rest (%)	93.1	2.8
TUG score (sec)	10.27	3.81
FTSST (sec)	18.96	10.63
Total CCQ score	3.6	0.9

FEV1 value – forced expiratory volume in 1 second, MRC - Medical Research Council Dyspnoea Scale, BMI – Body mass index, TUG - Timed Up & Go test, FTSST - The five times sit to stand test, CCQ - the Clinical COPD Questionnaire.

The group that dropped out during the intervention differed from the group that completed it. A larger proportion in the dropout group was men, a higher proportion received home help and they had poorer Borg scores at rest (4.5 vs. 2.6), FTSST (21.19 vs. 18.96 sec.) and poorer CCQ scores (4.09 vs. 3.58). There were no significant differences in the other clinical parameters.

### Clinical outcomes and safety

Table 2 shows the patients’ self-reported health status with COPD and physical performance before and after the intervention.

**Table 2:** **Changes in self-reported health status and physical performance from pre-intervention to post intervention**

**Variables**	**N**	**Pre-intervention**	**Post intervention**	**p-value**
TUG (sec)	37	9.35 (7.16;12.62)	8.34 (6.27;11.12)	<0.01
FTSST (sec)	37	16.61 (12.50;20.18)	12.94 (10.07;15.82)	<0.01
Total CCQ score	37	3.6 (3.2;4.3)	3.3 (2.5;3.6)	0.039

Data are medians (25th percentile; 75th percentile).

Analyses were performed with the use of related-samples Wilcoxon Signed Ranks Test. The significant level is 0.05. TUG - Timed Up & Go test, FTSST - The five times sit to stand test, CCQ - the Clinical COPD Questionnaire.

With regard to the safety of the patients there were no cases of falls or emergency contact with a general practitioner during the telemedicine training sessions.

### Patient perception

31 of 37 patients completing the programme submitted a postcard with information on their experience. The themes which emerged from the thematic analysis of patients’ responses can be divided into 3 broad areas: Proximity, accessibility and impact (see Table 3).

**Table 3:** **Themes emerged from the thematic analysis of patients’ postcard**

**Theme**	**Description**	**Tellingly quotes from postcards**
Proximity	The patient suitcase creates a notion of proximity. Patients experience that therapists are “with them” in their homes during telemedicine sessions, which provide reassurance. It also created space to train under safe conditions and confidence to do the exercises.	“*It is comforting to come home and have help by my side. All three weeks*.” (Patient No. 27).
		“*When the COPD suitcase was removed there was a void not to have to be ready at a given time*.” (Patient No. 24).
		“*It was a big help that she (the therapist) could see the exercises being done correctly*.” (Patient No. 44).
Access	The patient suitcase provides easy access to the supervised training and, in some cases, is the only way to get training. The intervention provides support to get started with being physically active, which for some patients is maintained after completing the intervention.	“*It was a good help to get started because when you are just sitting down by yourself, you will not get anything done. Especially when you can’t really get started after you have come home. So it’s smart to switch on the suitcase and get exercising.*” (Patient No. 37)
		“*I got more done when the therapist was there than when I was left alone with the exercises.*” (Patient No. 29)
		“*I got training and the desire to train. Without the suitcase I was unable to start. I got the motivation to train and use my body and I was also able to take the initiative to do it myself*.” (Patient No. 46)
Effect	Training and counselling via the patient suitcase is experienced to improve physical and mental capacity. Experiencing that exercise helps adhere to treatment which has a positive effect on health.	“It gave me renewed strength to start using my Flutter* 3 x a day some days more and to do some exercises. And now I’ve got more air.” (Patient No. 31).
		“*With training and counselling through the patient suitcase I have experienced that my general health has improved, I have more energy and on the days when my” breathing “is bad, it helps to train. It’s like something is “released” and I have extra space around the lungs and they are given more space/room for air.*” (Patient No. 50).

*Flutter: Mucus clearance device - a positive expiratory pressure (PEP) device.

### Economic aspects

Patients used the briefcase on average for 21 days. The hospital paid for renting the briefcases from the company (Medisat).

The use of hospital staff per patient is described in Table 4. Price per hour reflects the total expenditures to the hospital per staff member adjusted for sick leave, vacation and 1 hour for lunch, cleaning etc. Thus, price per hour is the price per hour of actual work with patients. When these expenditures are added to the expenditures of renting the briefcase the total expenditures per patient for the hospital can be estimated to be between € 852 and € 956 depending on whether the patients have 10 or 11 video calls. These are additional expenditures, since there are no expenditures of usual care because these patients declined to participate in rehabilitation at the hospital.

**Table 4:** **Expenditures of use of hospital staff per patient**

**Staff**	**Description**	**Minutes**	**Price per hour**	**Costs**
Physiotherapist	9 contacts, 20–30 min. each	180-270	€ 40	€ 121 - € 182
Occupational therapist	1-2 contacts, 60 min. each	60-120	€ 40	€ 40 - € 81
Secretary	2 times of 15 min.	70-75	€ 37	€ 43 - € 46
	8-9 times of 5 min.			
Total				€ 204 - € 309

Reimbursement to the hospital (if the Department is already producing the required quantity of treatments in accordance with the contract) corresponds to 90% of the DRG rate for highly specialized rehabilitation. For this type of rehabilitation, the DRG-tariff (GEN1A) is equal to € 97. Thus the total reimbursement to the hospital for 10 or 11 video consultations amounts to € 875 or € 963 retrospectively.

Comparison of the expenditures and the reimbursement show that if the number of video consultations per patient is on average 10 or 11, the reimbursement is slightly higher than the expenditures and thus the business case is positive.

### Organisational aspects

The focus group interview with two physiotherapists and one occupational therapist from the Department of Rehabilitation was carried out June 21th 2013. Findings revealed that: 1) There were very few technical problems and these had been solved in collaboration with the service provider, 2) No patient safety problems had been observed and no patients had fallen during the training sessions. This is expected to be a result of the physical tests carried out before the training was initiated, 3) More staff time in the ward was used due to the increased training, 4) Telemedicine training was seen to improve the rehabilitation program by improving the continuity between the hospital and the municipal services and 5) Telemedicine training gave the staff the opportunity to gain insight into a patient’s everyday life because they saw the patient dressed in his or her own clothing and in his or her own home and surroundings: a physiotherapist with experience in telemedicine training commented “*Now the patients look like real human beings*”.

## Discussion

This study has shown how telemedicine can support rehabilitation of patients with severe COPD, who are not able (or willing) to participate in established rehabilitation programmes provided by hospitals and municipal training centres. Results from the study show that telemedicine training and counselling aimed at improving functioning can be carried out under safe conditions and are likely to have an impact on clinical outcomes in patients with severe COPD just after hospitalization with an exacerbation of COPD.

The experiences from this study suggests that the Patients Briefcase, monitoring provided using pulse oximetry and a physiotherapist, were sufficient to allow safe and effective supervised exercise training for patients with severe COPD at home. In this study the programme completion rate was higher than in the Holland et al. study [11], but in the Holland et al. study the programme lasted for 8 weeks compared to our 3 week programme. For the participants who completed the programme, we found substantial improvements in FTSST. We know from other studies that resistance training can improve muscle strength in patients with exacerbation of COPD [27-29] even after short training sessions over 7 days, with a load of 70% of 1RM [29].

This study was not conducted with a randomized controlled design; we therefore cannot say whether the achieved post intervention effect can be attributed to the intervention or whether other factors have played a role, such as improvement in the inflammatory condition. A recently study did not find effect of early rehabilitation intervention to enhance recovery during hospital admission for an exacerbation of chronic respiratory disease, which might indicate that the participant recovered naturally, but as it was unclear if the intervention was sufficiently intense [30], there is still need for studies that examine the efficacy of supervised physical training after hospitalization with exacerbation. With a dropout rate of 25% and no intention to treat, analysis bias associated with the results cannot be ruled out. In the rehabilitation of patients with COPD, the supervised exercise training is recommended to last at least six to eight weeks with twice-weekly sessions [2]. Therefore, this individual 3-week supervised exercise program for patients with severe COPD will in most cases not be sufficient. Currently, patients are referred to individualized rehabilitation in the municipality after the telemedicine training. Our study suggests that telemedicine can be a useful element in the rehabilitation of patients with COPD who declines out-patient rehabilitation. There is a need for further investigation into how a supervised home-based training program for patients with severe COPD should be planned and for exploration of how telemedicine rehabilitation may be effective for health improvements in patients with severe COPD with an exacerbation of their condition. A previous study that tested the effect of telecounselling by a nurse using the patient briefcase found the equipment easy to use, technically stable with high up-time, high confidence in the equipment from nursing staff and high patient satisfaction [31]. Using satellite involved a delay, which was slightly disturbing, but could be dealt with through training [31].

In this study, health status was assessed by the CCQ. The CCQ has shown good reliability, validity, and responsiveness at the group and individual levels in patients with COPD [32]. CCQ was also found to be a predictor of mortality in patients with COPD, and as mortality and health status are important clinical endpoints, it has been suggested that CCQ could be used to target interventions [33]. CCQ was found to correlate with St. George’s Respiratory Questionnaire (SGRQ) and The COPD Assessment Test (CAT) in patients with severe COPD undergoing pulmonary rehabilitation [34]. SGRQ, that includes 50 questions, is commonly used in the assessment of health status in pulmonary rehabilitation trials [2,32], but we did not find SGRQ feasible for use in our study because the participants had to complete the questionnaire at home without assistance. Ringbaek et al. found that the need for assistance while answering the questionnaire was 86.5% for SGRQ, 53.9% for CAT, and 36.0% for CCQ [34]. All the CCQ questionnaires received from the participants in our study were completed. The functional test assessing physical performance was performed in patients’ own homes. It was not possible to perform the shuttle walk test or the 6 minute walk test in the patients’ homes because of lack of space. These two tests are commonly used to measure physical performance in the evaluation of physical training in patients with COPD [3]. A sit-to-stand test (FTSST) was used to measure functional lower limb muscle strength in this study. The FTSST has previously been found to be reliable, valid and responsive in patients with COPD and suitable for use in most healthcare settings [35]. The sit-to-stand test is associated with mortality [36] and strength of the quadriceps muscle [37] in patients with COPD. Physical performance tests are important for characterizing COPD patients and predicting their prognosis [36], in our study we found FTSST easy to administer, feasible and easy to apply in the patients’ homes. The timed Up & Go test (TUG) was found to be feasible and easy to apply in the patients’ homes in this study. This test has been found useful to explore functional balance impairment among older adults with COPD [38], and our study suggest that TUG could be used to complement other physical performance tests when evaluating physical training of people with severe COPD.

This study showed that it is important for patients with severe COPD to have access to supervised training and counselling. The study participants believed this to be essential for getting started with being physically active as well as for improving their physical and mental wellbeing. The participants’ connection via the Patient Briefcase provided them with a feeling of proximity and security when in contact with their therapists. A smaller study, which tested an internet-based rehabilitation programme including supervised training for COPD patients, showed that the intervention was well received by the participants [39]. Another study of home-based exercise rehabilitation with telemedicine following cardiac surgery scored very highly for health professional support, yield/effect, and ease of use [40]. These findings are supported by a systematic review, which shows that patients with severe COPD experience trust and peace of mind when receiving healthcare via telemedicine solutions [41]. The review does not specifically focus on telemedicine training but includes all types of telemedicine treatment. The postcard with the open ended question gave access to varied knowledge about the individual experiences and opinions about the intervention, but due to the nature of this way of gaining information we were not able to ask deeper into specific answers. An in-depth interview would have given a more exhaustive knowledge about the patient perspective as it gives the individual the opportunity to develop and give reasons for his or hers point of views.

The business case indicated that under the current contract financing system and DRG-rates, the reimbursement to the hospital is slightly higher than the hospital expenditures. Thus the business case for the hospital was positive, also compared to usual care because these patients have declined rehabilitation at the hospital. Two main factors may influence this result. Firstly, the registration of the treatments in the administrative system must be correct. Secondly, the compliance of the patients and their participation for the whole three weeks are important. If the patients only have five consultations, the expenditures remain about the same, but the reimbursement will be only half as much. It should be noted, that data on use of staff is uncertain because it was based on an assessment made by the project manager after the pilot study and that the results need to be confirmed in a larger prospective study. In addition the business case performed has a very narrow perspective on the expenditures and reimbursement for the hospital and did not include the wider societal costs or the cost-effectiveness of the intervention.

The focus group interviews showed that the clinicians found the safety of the service acceptable, that the service improved the continuity of the rehabilitation programme for the patients and that the service improved the possibility of including the patient’s everyday life by giving the staff the opportunity to see the patient in his or hers own home. The total number of therapists involved in the project was four, but only three participated in the focus group interview because one therapist was no longer working at the hospital at the time. In addition the interview was done six months after the study period and this may have affected the answers from the clinicians.

## Conclusions

This study showed that home-based supervised training and counselling via video conference is feasible with regard to clinical outcomes, safety and patient perception. The study was designed to benefit patients who declined to participate in the established rehabilitation programmes in the hospital. This means that telemedicine can help to ensure more equitable access to supervised training for patients with severe COPD. The study had some limitations: 1) patients who were included in this study represent a sample of the poorest patients with severe COPD, but not those patients with the most serious comorbidity. Comorbidity is the cause of many COPD patients’ deaths. It might be appropriate to examine telemedicine rehabilitation programme for patients with many comorbidities; 2) the interpretation of results of clinical outcomes is limited by the absence of a control group. Nevertheless, it seems that this type of rehabilitation programme could usefully be implemented for selected patient groups, where distance from a clinic/hospital is a problem for maintaining progress. We would recommend a study with a randomized design that examines the effects of telemedicine rehabilitation including supervised training of patients with severe COPD. When researching the development of telemedicine solutions, it is important to consider the technical solution and the service simultaneously, what contribution telemedicine makes and to explore how each service is used by the relevant users and how it fits into the individual’s daily practice. Therefore, it is essential that future research includes clinical, patient-centred, economic and organizational perspectives [42,43].

## Abbreviations

COPD: Chronic obstructive pulmonary disease
ADSL: Asymmetric Digital Subscriber Line
MAST: Model for assessment of telemedicine
FEV1 value: Forced expiratory volume in 1 second
FVC: Forced vital capacity
MRC: Medical Research Council Dyspnoea Scale
BP: Blood pressure
AMI: Acute myocardial infarction (AMI)
EF: Ejection fraction
RM: Repetitions Maximum
COPM: The Canadian Occupational Performance Measure
CCQ: The Clinical COPD Questionnaire (CCQ)
TUG: Timed Up & Go test
FTSST: The five times sit to stand test
DRG: Diagnose Related Groups
DKR: Danish krone
SPSS: Statistical Package for the Social Sciences
BMI: Body Mass Index
SGRQ: St. George’s Respiratory Questionnaire
CAT: The COPD Assessment Test
